# A Case of Hysterical Photophobia in a Child

**Published:** 1910-10-22

**Authors:** A. C. D. Firth

**Affiliations:** Victoria Hospital for Children, Chelsea.


### The General Practitioner's Column.

[Contributions to this Column are invited, and, if accepted, will be paid for.]

### A CASE OF HYSTERICAL PHOTOPHOBIA IN A CHILD.

By A. C. D. FIRTH, B.C. (Cantab), R.M.O., Victoria Hospital for Children, Chelsea.

It is not always an easy matter to differentiate between real disease and hysteria, even when the age and sex of the patient favour the latter condition. In the case recorded here, once suspicion was aroused, the diagnosis became easy, but the unusual form, and early age at first directed the train of thought to the probability of organic disease rather than to hysterical simulation.

The child, a girl aged eight years, was brought to me for "photophobia" of three days' duration, coming on suddenly after a day's work at school. She kept her eyes tightly closed, and when asked to open them only did so very slightly and in the peculiar blinking manner common in children with some disease of the cornea. Nothing abnormal was detected in the eyes, so the case was referred to the ophthalmic surgeon. He examined the eyes and returned a report to the effect that both media and fundi appeared to be perfectly healthy.

On this information the possibility of hysterical simulation suggested itself, and when the application of a spatula to the uvula and soft palate failed to provoke the usual response, the case at once took a different aspect, confirmatory evidence being supplied by the reduplicated knee-jerks. The child was admitted to the hospital, and remained in the "photophobic" condition for some twenty-four hours. At the expiration of that time a dose of castor-oil was ordered for the usual reason. The result was surprising, but unintentional.

The next morning the child was sitting up in bed, gazing open-eyed around the ward, and, in reply to a question, stated that after the medicine of the previous evening she found, on waking, she could open her eyes. She was discharged soon afterwards, with no symptoms of ocular trouble remaining, but the palatal reflex was still absent, and the knee-jerks very brisk. The case in itself is trivial, although one from which much kudos might have resulted in another century when a belief in miracles was not lacking; but it serves to emphasise the fact that a condition which in children is usually the result of organic disease may be simulated in the age of innocence.

